# Evaluation of Low- and Middle-Income Country Authorship in the Global Orthopaedic Literature

**DOI:** 10.5435/JAAOSGlobal-D-22-00168

**Published:** 2023-02-17

**Authors:** Jason Young, Rachel Chen, Soyoun Choi, Ian B. Hayes, Paul A. Bain, Collin May

**Affiliations:** From the Harvard Combined Orthopedic Residency Program, Boston, MA (Dr. Young); the Harvard College, Boston, MA (Ms. Chen, Ms. Choi, and Mr. Hayes); the Harvard Medical School, Boston, MA (Dr. Bain and Dr. May); the Countway Library of Medicine, Harvard Medical School, Boston, MA (Dr. Bain); and the Boston Children's Hospital, Boston, MA (Dr. May).

## Abstract

Extensive research collaborations exist between high-income countries and low- and middle-income countries (LMICs), although prior work has raised concerns regarding equitable representation among LMIC authors. The goal of this bibliometric analysis was to characterize LMIC authorship among indexed orthopaedic journals and identify factors contributing to disparities in representation.

**Methods:**

We identified all articles appearing in orthopaedic journals indexed in MEDLINE and Journal Citation Reports with a focus on LMICs or cohorts between 2009 and 2018. All articles describing research conducted in LMICs or research focused on applications to cohorts in LMIC(s) were included. Author affiliation, article characteristics, and impact factor were assessed for 1,573 articles. Logistic regression models created to identify predictors of LMIC authorship.

**Results:**

We identified few studies published in indexed journals focused exclusively on LICs. Funded studies were less likely to have LMIC last authors. Compared with articles published in lower impact factor journals, those in journals with a higher impact factor were less likely to have a LMIC first or last author. The greater the number of countries represented per study, the less likely it had a LMIC first or last author.

**Conclusion:**

Our study highlights persistent disparities in authorship from LMICs in indexed orthopaedic journals.

The importance of building global health research capacity in low- and middle-income countries (LMICs) is being recognized more broadly.^1,2^ The number of research collaborations between high-income countries (HICs) and LMICs has been increasing over time.^3^ However, these efforts have led to disparities in professional recognition in the publication of global health research.^1,2,4,5^ Prior work has indicated that authors from LMICs are underrepresented as first and last authors on studies pertaining to local cohorts,^1,6^ and several studies have raised concerns regarding the agency of local partners in global health collaborations and fairness of partnerships therein.^7,8^

As such, disparities in authorship equity and representation pose a challenge both to parity in research collaborations and to the establishment of robust local research infrastructures. Several studies have proposed potential causes of authorship underrepresentation for researchers from LMICs, including language barriers,^9^ lack of awareness among local researchers regarding criteria to be listed as authors on academic studies,^8,9^ inequities in funding,^9^ and the perpetuation of power structures that benefit the HICs at the expense of local researchers.^2,10^

Although prior work on this subject has focused on traditional subjects in the global health landscape, such as in HIV, malaria, or tuberculosis,^4^ no analysis has thus far been conducted on authorship within the international orthopaedic or global health–focused orthopaedic literature. Prior bibliometric studies of the global surgery landscape^11^ and LMIC orthopaedic research output^12^ exist, although a dedicated analysis of authorship has yet to be performed in global orthopaedics.

This study presents a bibliometric analysis assessing authorship of health-related studies conducted in LMICs and published in indexed orthopaedic journals. Specifically, we seek to better characterize LMIC authorship within orthopaedics to better inform global orthopaedic research capacity building efforts and encourage more equitable research practices.

## Methods

### Journal Search Methods

The National Library of Medicine Catalog was queried on May 15, 2020, for all journals indexed in MEDLINE under Medical Subject Headings terms relating to orthopaedic surgery (Supplemental File 1, http://links.lww.com/JG9/A264). MEDLINE represents the portion of PubMed records indexed with Medical Subject Headings terms. A description of the MEDLINE journal selection process is provided in Supplemental File 2, http://links.lww.com/JG9/A265. Based on our search, no journals were identified that were previously indexed in MEDLINE that were not indexed currently. As such, all MEDLINE journals were included in article searches for subsequent years.

Journal Citation Reports was also queried on May 15, 2020, in our initial journal list generation (jcr.clairvate.com). Journal Citation Reports (Clarivate Analytics, Philadelphia, USA) provides analytics on academic journals through an annual report across various scientific disciplines.^13^ A description of journal selection for JCR is provided in Supplemental File 3, http://links.lww.com/JG9/A266. Journal Citation Reports for all journals indexed under the “Orthopedics” subject term for each year 2009 to 2018 (inclusive) were collected.

**Figure 1:**
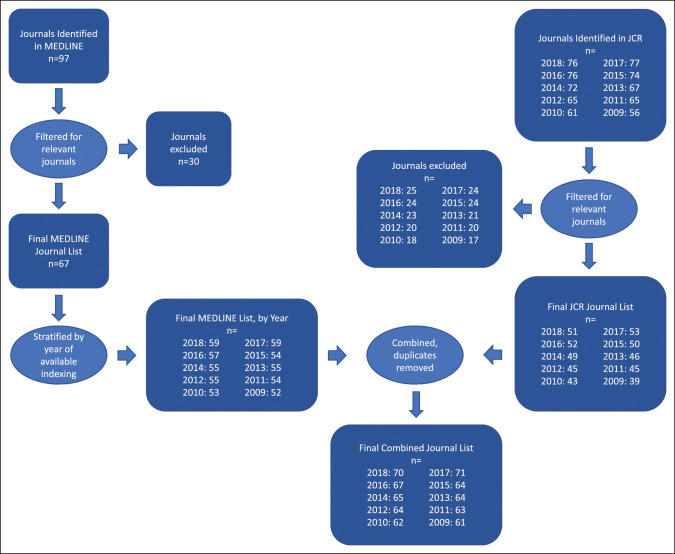
Journal selection methods. JCR = Journal Citation Reports.

Journal lists from MEDLINE and JCR were subsequently evaluated by one author (J.Y.), and any journals that did not have a primary focus in clinical management of orthopaedic surgical conditions were excluded. Journals identified in MEDLINE were subsequently evaluated by year for our study period and included in our search only if indexed for the year in question. A final unique list of journals stratified by year was then constructed (Figure 1). All journals were then evaluated for indexing in PubMed for each year in our study period. Journals from both MEDLINE and JCR that were indexed in PubMed were subsequently searched using the latter search engine. Articles from journals uniquely listed in JCR (n = 4) were searched separately in Web of Science.

### Article Search Methods

**Figure 2:**
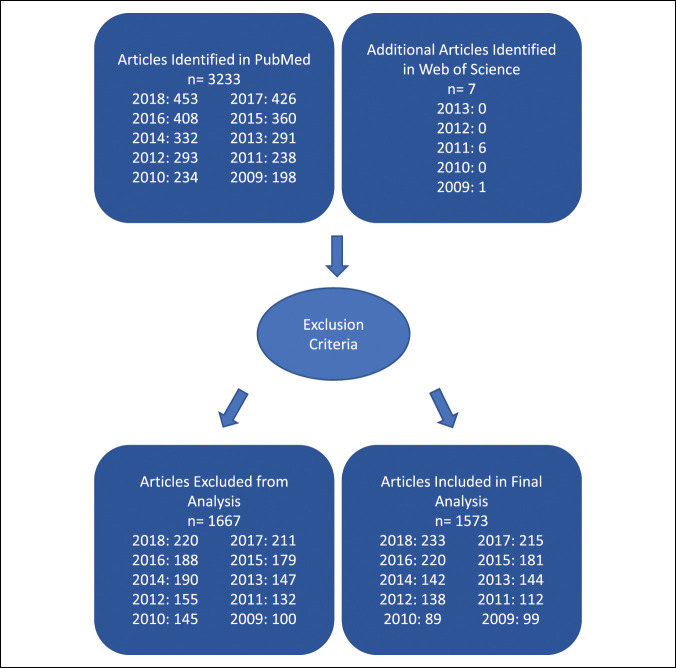
Article search methods.

For all journals indexed in PubMed for a given year, titles and abstracts were searched in PubMed (https://pubmed.ncbi.nlm.nih.gov/) on June 1, 2020, for mention of a LMIC or associated keyword (Supplemental File 4, http://links.lww.com/JG9/A267). Similar methods were used for journals uniquely listed in Web of Science (Supplemental File 5, http://links.lww.com/JG9/A268) to obtain a final article list. (Figure 2).

### Inclusion Criteria

All articles from the above journals describing (1) research conducted in LMICs or (2) research focused on applications to cohorts in LMIC(s) were included in this study. We sought to include all studies' international collaborative work involving or focused on LMICs, and as such, studies conducted in HICs or LMICs were both included, so long as they fulfilled the above criteria. A study's country of focus was abstracted from the study text. If the study location was not apparent from the article text, the country was assumed if all listed authors had affiliations within a single country. In cases where multinational authors conducted a study for which region or country was not clear, the corresponding author of the said study was contacted through e-mail for clarification.

### Exclusion Criteria

Studies conducted in HICs and not pertaining to LMICs were excluded from this study. All editorials, abstract/meeting summaries, narrative pieces, obituaries, or opinion pieces were also excluded. Links to erratum were excluded, but the associated articles were evaluated for eligibility.

### Data Collection and Analysis

Each article abstract was evaluated by two of three authors (R.C., S.C., or I.H.) for eligibility. Study information, including year of publication, country of author affiliation, funding information, open access status, impact factor, number of authors, number of countries represented, and study type, was recorded for each eligible study. Authors' country of affiliation was collected based on listed affiliation from the article title and text, and income status of the country (HIC, MIC, or LIC) was extrapolated from its World Bank Country Income Classification status.^14^ We chose to tackle the question of research representation through the lens of authorship because it serves as a proxy for available research opportunities as well as research leadership for the authors involved, as has been previously supported in the literature.^4^ We chose to focus particularly on first and last authorship because these have been considered positions of leadership for published research, and prior work on this topic has used this accepted convention.^1,6^ If an author had affiliations with both HICs and LMICs, he or she was assigned a HIC affiliation, as has been done in prior bibliometric studies.^4^ The rationale for this decision in our study rests on the theoretical resource advantages a HIC affiliation would provide. If an author had more than one LMIC affiliation, the first listed affiliation was assumed to be the primary affiliation. Funding type was coded as one of six categories: academic, HIC governmental funding, LMIC governmental funding, NGO funding, private industry funding, or funding from multiple/mixed sources. Study type was coded as one of five categories: clinical, preclinical or translational, epidemiologic or economic, global health focused (studies about or related to improving health care or healthcare capacity in low-resource settings), or focused on medical education.

Authors R.C., S.C., and I.H. were trained on categorizing the 2018 PubMed data set, and all data collection was evaluated by a second author (J.Y.) for accuracy. Errors were corrected, and the data collection tool was adjusted before beginning complete data collection for the entire data set. In ambiguous cases or in cases where two of the above authors disagreed about eligibility or categorization, a third author (J.Y.) decided on exclusion/inclusion or accurate categorization.

Statistical analyses were performed in SAS (SAS, Cary, NC). Differences in study characteristics were evaluated with chi-square tests. Final models were constructed to evaluate for predictors of LMIC first and last authorship using logistic regression models. Alpha was set at 0.05.

## Results

In total, 3,240 articles were identified, of which 1,573 met inclusion criteria for this study (Figure 2). The number of studies included per country is presented in Supplemental Table 1, http://links.lww.com/JG9/A259. Of the 1573 studies included, 1412 (89.8%) had a LMIC first author. The full presentation of article characteristics for first author studies is presented in Table 1. Of the studies included in this analysis, LMIC first authors comprised 93 of 95 (97.9%) preclinical or translational science studies, 1043 of 1231 (84.7%) clinical research studies, 148 of 175 (84.6%) epidemiologic and economic studies, 5 of 45 (11.1%) global health studies, and 23 of 27 (85.2%) medical education studies. LMIC first authors accounted for 281 of 339 (82.9%) funded studies and 1131 of 1234 (91.7%) unfunded studies. When examining the impact factor, LMIC first authors represented 251 of 264 (95.1%) studies without an assigned impact factor, 380 of 390 (97.4%) studies with an impact factor of <1, 389 of 454 (85.7%) studies with an impact factor between 1 and 2, 316 of 370 (85.4%) studies with an impact factor between 2 and 3, and 76 of 95 (80.0%) studies with an impact factor above 3. Similar article characteristics for last authors are presented in Table 2.

**Table 1:** Study Characteristics by LMIC First Authorship

Study Characteristics	No. of Articles With LMIC First Author/All Publications	LMIC Author Proportion
Total	1412/1573	89.8
Year of publication		
2009	89/99	89.9
2010	84/89	94.4
2011	106/112	94.6
2012	127/138	92.0
2013	132/144	91.7
2014	124/142	87.3
2015	161/181	89.0
2016	200/220	90.9
2017	197/215	91.6
2018	192/233	82.4
Study type		
Preclinical or translational science	93/95	97.9
Clinical	1043/1231	84.7
Epidemiologic and economic analyses	148/175	84.6
Global health interventions	5/45	11.1
Medical education	23/27	85.2
Article type		
Open access	743/813	91.4
Subscription access	669/760	88.0
Funding		
Yes	281/339	82.9
No	1131/1234	91.7
Funding type		
Academic	58/61	95.1
HIC government	0/7	0.0
LMIC government	154/157	98.1
Multiple-mixed	41/59	69.5
NGO	2/6	33.3
Private/industry	26/49	53.1
Impact factor		
N/A	251/264	95.1
<1	380/390	97.4
1-2	389/454	85.7
2-3	316/370	85.4
3+	76/95	80.0
No. of authors per study		
1	33/38	86.8
2	98/105	93.3
3	158/169	93.5
4	203/224	90.6
5+	920/1037	88.7
No. of countries in the author affiliations		
1	1317/1389	94.8
2	78/126	61.9
3	9/30	30.0
4	5/13	38.5
5+	3/15	20.0

HIC = high-income country, LMIC = low- and middle-income country, N/A = not applicable

**Table 2:** Study Characteristics by LMIC Last Authorship

Study Characteristics	No. of Articles With LMIC Last Author/All Publications	LMIC Author Proportion
Total	1348/1535	87.8
Year of publication		
2009	89/97	91.8
2010	80/82	97.6
2011	102/107	95.3
2012	120/131	91.6
2013	128/140	91.4
2014	118/142	83.1
2015	153/180	85.0
2016	195/216	90.3
2017	182/213	85.4
2018	181/227	79.7
Study type		
Preclinical or translational science	87/95	91.6
Clinical	1093/1200	91.1
Epidemiologic and economic analyses	140/172	81.4
Global health interventions	8/44	18.2
Medical education	20/24	83.3
Article type		
Open access	721/796	90.6
Subscription access	627/739	84.8
Funding		
Yes	271/339	79.9
No	1077/1196	90.1
Funding type		
Academic	52/61	85.2
HIC government	1/7	14.3
LMIC government	156/157	99.4
Multiple-mixed	40/59	67.8
NGO	2/6	33.3
Private/industry	20/49	40.8
Impact factor		
N/A	234/251	93.2
<1	366/380	96.3
1-2	367/445	82.5
2-3	307/364	84.3
3+	74/95	77.9
No. of authors per study		
2	94/105	89.5
3	156/169	92.3
4	202/224	90.2
5+	896/1037	86.4
No. of countries in the author affiliations		
1	1284/1351	95.0
2	50/126	39.7
3	5/30	16.7
4	3/13	23.1
5+	6/15	40.0

HIC = high-income country, LMIC = low- and middle-income country, N/A = not applicable

First and last authorship characteristics stratified by LIC and MIC are presented in Tables 3 and 4, respectively. Studies without a single country of focus (multinational studies or studies focused on LMICs in general) were not included in this stratification. For studies conducted in or focused on MICs, 1371 of 1422 (96.4%) studies had a LMIC first author, whereas for LICs, 23 of 52 (44.2%) had a LMIC first author. LMIC last authors comprised 1315 of 1389 (94.7%) studies from MICs and 18 of 52 (34.6%) from LICs. Of studies with an impact factor of three or greater, seven studies were conducted in or focused on LICs, whereas 78 were conducted in MICs. Of this subset, 4 of 7 (57.1%) studies from LICs had a LMIC first author, whereas 1 of 7 (14.3%) had a LMIC last author. For MIC studies, 71 of 78 (91.0%) had a LMIC first author, whereas 73 of 78 (93.6%) had a LMIC last author.

**Table 3:** Study Characteristics by LMIC First Authorship Stratified by World Bank Country Type

Study Characteristics	LIC Study Countries		MIC Study Countries	
	No. of Articles With LIC First Author/All Publications	Authorship Proportion (%)	No. of Articles With MIC First Author/All Publications	Authorship Proportion (%)
Total	23/52	44.2	1371/1422	96.4
Year of publication				
2009	1/4	25.0	88/93	94.6
2010	1/1	100.0	83/83	100.0
2011	2/2	100.0	104/104	100.0
2012	2/2	100.0	125/128	97.7
2013	1/1	100.0	130/133	97.7
2014	2/10	20.0	121/127	95.3
2015	6/13	46.2	154/160	96.3
2016	2/5	40.0	194/202	96.0
2017	3/3	100.0	189/200	94.5
2018	3/11	27.3	183/192	95.3
Study type				
Preclinical/translational	1/1	100.0	91/92	98.9
Clinical	18/27	66.7	1109/1138	97.5
Epidemiologic and economic analyses	1/6	16.7	147/156	94.2
Global health interventions	3/18	16.7	1/12	8.3
Medical education	0/0	—	23/24	95.8
Article type				
Open access	11/23	47.8	725/757	95.8
Subscription access	12/29	41.4	646/665	97.1
Funding				
Yes	5/12	41.7	271/293	92.5
No	18/40	45.0	1100/1129	97.4
Funding type				
Academic	1/1	100.0	57/58	98.3
HIC government	0/1	0.0	0/3	0.0
LMIC government	0/0	—	154/157	98.1
Multiple-mixed	0/1	0.0	40/49	81.6
NGO	0/0	—	2/3	66.7
Private/industry	4/9	44.4	18/23	78.3
Impact factor				
N/A	3/4	75.0	248/255	97.3
<1	3/6	50.0	373/375	99.5
1-2	6/21	28.6	378/398	95.0
2-3	7/14	50.0	301/316	95.3
3+	4/7	57.1	71/78	91.0
No. of authors per study				
1	0/0	—	33/33	100.0
2	3/4	75.0	95/97	97.9
3	4/6	66.7	153/157	97.5
4	4/10	40.0	195/201	97.0
5+	12/32	37.5	895/934	95.8
No. of countries in the author affiliations				
1	13/22	59.1	1298/1316	98.6
2	9/24	37.5	64/86	74.4
3	1/6	16.7	7/15	46.7
4	0/0	—	1/3	33.3
5+	0/0	—	1/2	50.0

LMIC = low- and middle-income country

**Table 4:** Study Characteristics by LMIC Last Authorship Stratified by World Bank Country Type

Study Characteristics	LIC Study Countries		MIC Study Countries	
	No. of Articles With LIC Last Author/All Publications	Authorship Proportion (%)	No. of Articles With MIC Last Author/All Publications	Authorship Proportion (%)
Total	18/52	34.6	1315/1389	94.7
Year of publication				
2009	3/4	75.0	86/91	94.5
2010	1/1	100.0	79/79	100.0
2011	2/2	100.0	100/100	100.0
2012	2/2	100.0	118/121	97.5
2013	1/1	100.0	125/129	96.9
2014	2/10	20.0	116/127	91.3
2015	4/13	30.8	148/159	93.1
2016	2/5	40.0	188/198	94.9
2017	0/3	0.0	181/199	91.0
2018	1/11	9.1	174/186	93.5
Study type				
Preclinical/translational	0/1	0.0	87/92	94.6
Clinical	13/27	48.1	1066/1110	96.0
Epidemiologic and economic analyses	0/6	0.0	140/154	90.9
Global health interventions	5/18	27.8	2/12	16.7
Medical education	0/0	—	20/21	95.2
Article type				
Open access	10/23	43.5	705/741	95.1
Subscription access	8/29	27.6	610/648	94.1
Funding				
Yes	3/12	25.0	262/293	89.4
No	15/40	37.5	1053/1096	96.1
Funding type				
Academic	0/1	0.0	52/58	89.7
HIC government	0/1	0.0	1/3	33.3
LMIC government	0/0	—	156/157	99.4
Multiple-mixed	1/1	100.0	38/49	77.6
NGO	0/0	—	2/3	66.7
Private/industry	2/9	22.2	13/23	56.5
Impact factor				
N/A	2/4	50.0	231/242	95.5
<1	1/6	16.7	363/366	99.2
1-2	7/21	33.3	358/392	91.3
2-3	7/14	50.0	290/311	93.2
3-4	1/7	14.3	73/78	93.6
No. of authors per study				
2	1/4	25.0	93/97	95.9
3	3/6	50.0	152/157	96.8
4	4/10	40.0	195/201	97.0
5+	10/32	31.3	875/934	93.7
No. of countries in the author affiliations				
1	13/22	59.1	1265/1283	98.6
2	5/24	20.8	42/86	48.8
3	0/6	0.0	5/15	33.3
4	0/0	—	2/3	66.7
5+	0/0	—	1/2	50.0

HIC = high-income country, LMIC = low- and middle-income country, N/A = not applicable

Multivariable regression analyses are presented in Table 5. Odds ratios (ORs) were calculated for the likelihood of LMIC first and last authorship based on the predictor variables outlined above. More recent studies were more likely to have LMIC first authorship, OR = 1.09 ([CI: 1.00, 1.19], *P* = 0.0416), but less likely to have LMIC last authorship, OR = 0.93 ([CI: 0.86, 0.99], *P* = 0.0382). Studies that received funding were less likely to have LMIC last authors, OR = 0.62 ([CI: 0.41, 0.96], *P* = 0.0297). Compared with clinical studies, LMIC first authorship was more likely for preclinical and translational science papers, OR = 4.41 ([CI: 1.00, 9.39], *P* < 0.0001), and less likely for epidemiologic and economic analyses, OR = 0.37 ([CI: 0.22, 0.63], *P* = 0.007); global health studies, OR = 0.01 ([CI: 0.01, 0.03], *P* < 0.001); and medical education studies, OR = 0.26 ([CI: 0.08, 0.84], *P* = 0.0065). Similarly, LMIC last authorship was also less likely for epidemiologic and economic analyses, OR = 0.35 ([CI: 0.21, 0.56], *P* = 0.0079), and global health studies, OR = 0.03 ([CI: 0.01, 0.06], *P* < 0.0001). Compared with studies published in journals with an impact factor of <1, studies published in journals with a higher impact factor were less likely to have a LMIC first author {IF 1-2: OR = 0.08 ([CI: 0.08, 0.39], *P* = 0.0005); IF 2-3: OR = 0.22 ([CI: 0.10, 0.51], *P* = 0.0434); IF 3+: OR = 0.24 ([CI: 0.08, 0.72], *P* = 0.0351)}. Similar findings were observed for LMIC last authors as well {IF 1-2: OR = 0.20 ([CI: 0.11, 0.38], *P* < 0.0001); IF 2-3: OR = 0.31 ([CI: 0.16, 0.63], *P* = 0.0032); IF 3+: OR = 0.37 ([CI: 0.15, 0.89], *P* = 0.0434)}. Studies with more authors were more likely to have a LMIC first author, OR = 1.08 ([CI: 1.01, 1.15], *P* = 0.0207), and a LMIC last author, OR = 1.25 ([CI: 1.15, 1.36], *P* < 0.0001). Finally, the greater the number of countries represented in the author affiliations, the less likely there was a LMIC first author, OR = 0.20 ([CI: 0.15, 0.28], *P* < 0.0001), or last author, OR = 0.32 ([CI: 0.24, 0.42], *P* < 0.0001).

**Table 5:** Predictors of LMIC First Authorship/LMIC Last Authorship

Variable	Predictors of LMIC First Authorship (N = 1573)		Predictors of LMIC Last Authorship (N = 1535)	
	aOR (95% CI)	*P*	aOR (95% CI)	*P*
Year	1.09 (1.00, 1.19)	0.0416	0.93 (0.86, 0.99)	0.0382
Article type				
Subscription access	1.0 (reference)		1.0 (reference)	
Open access	0.92 (0.59, 1.43)	0.7207	1.34 (0.91, 1.97)	0.1346
Study type				
Clinical	1.0 (reference)		1.0 (reference)	
Preclinical or translational science	4.41 (1.00, 9.39)	<0.0001	1.08 (0.48, 2.42)	0.1009
Epidemiologic and economic analyses	0.37 (0.22, 0.63)	0.0070	0.35 (0.21, 0.56)	0.0079
Global health interventions	0.01 (0.01, 0.03)	<0.0001	0.03 (0.01, 0.06)	<0.0001
Medical education	0.26 (0.08, 0.84)	0.0065	0.43 (0.13, 1.40)	0.6099
Funding				
No	1.0 (reference)		1.0 (reference)	
Yes	0.70 (0.43, 1.15)	0.1623	0.62 (0.41, 0.96)	0.0297
Impact factor				
<1	1.0 (reference)		1.0 (reference)	
1-2	0.18 (0.08, 0.39)	0.0005	0.20 (0.11, 0.38)	<0.0001
2-3	0.22 (0.10, 0.51)	0.0434	0.31 (0.16, 0.63)	0.0032
3+	0.24 (0.08, 0.72)	0.0351	0.37 (0.15, 0.89)	0.0434
N/A	0.46 (0.18, 1.19)	0.2755	0.55 (0.25, 1.21)	0.2523
No. of authors per study	1.08 (1.01, 1.15)	0.0207	1.25 (1.15, 1.36)	<0.0001
No. of countries in the author affiliations	0.20 (0.15, 0.28)	<0.0001	0.32 (0.24, 0.42)	<0.0001

^a^OR = adjusted odds ratio, LMIC = low- and middle-income country

Adjusted odds ratio derived from logistic regressions.

## Sensitivity Analyses

The accuracy of reported author affiliation in clinical research is not well evaluated, and the degree of inaccurate affiliations is unknown.^4^ Consequently, post hoc sensitivity analyses were performed to assess the robustness of reported findings and account for possible error in authorship country of affiliation using the Wald test of joint significance, as previously described.^4^ Results are reported in Supplemental Figures 1–4, http://links.lww.com/JG9/A269, and Supplemental Tables 2–5, http://links.lww.com/JG9/A260, http://links.lww.com/JG9/A261, http://links.lww.com/JG9/A262, http://links.lww.com/JG9/A263. Our findings were robust at 10% authorship misclassification, with exception of the findings that the LMIC first authorship likelihood is higher with preclinical/translational science studies and lower with medical education studies, the findings of a higher impact factor being associated with lower LMIC first and last authorship for studies in journals with IF >2, and the finding that there was an increased likelihood of LMIC first authorship with an increasing number of total study authors.

## Discussion

Our study is, to our knowledge, the first to characterize authorship from LMICs in indexed orthopaedic journals on research focused on LMICs. We identified relatively few studies published in indexed journals focused exclusively on LICs. Funded studies were less likely to have LMIC last authors. Compared with clinical studies, LMIC first authorship was more likely for preclinical and translational science research and less likely for epidemiologic and economic analyses, medical education studies, and global health studies. Similarly, LMIC last authorship was also less likely for epidemiologic and economic analyses and for global health studies. Compared with articles published in lower impact factor journals, those in higher impact factor journals were less likely to have LMIC first or last authors. The greater the number of countries represented per study, the less likely the study was to have a LMIC first or last author. Studies with more authors were more likely to have a LMIC first or last author. Although more recent studies were more likely to have LMIC first authors, they were also less likely to have LMIC last authors.

Excluding studies with a multinational focus, we identified only 52 studies that were based in or principally concerning LIC, of which 23 of 52 had a LMIC first author (44.2%) and 18 of 52 (34.6%) had a LMIC last author (Tables 3 and 4). By contrast, most studies published in MICs included in our analysis had a LMIC first 96.4% (1371/1422) or last 94.7% (1315/1389) author. The differential rates of LIC and MIC authorship mirror trends in the broader global health literature.^7-9,15,16^ Although the exact reasons for low levels of LIC authorship remain unclear, prior work has posited several hypotheses, including scarcity of funding from LMICs,^6,17^ language barriers,^17^ poor understanding of authorship criteria among LMIC authors,^8,17^ insufficient research capacity to conduct higher level studies,^6^ differences in cultural values,^17^ and the practice of safari research, where studies are conducted and driven by HIC authors, and LMIC involvement is nominal in nature to boost a study's credibility.^6^

Compared with studies published in journals with an impact factor of <1, studies published in journals with a higher impact factor were less likely to have LMIC first authors or LMIC last authors. In addition, among studies from LICs, few studies published in high-impact journals were identified (n = 7), of which only 4 of 7 (57.1%) had a LMIC first author, whereas 1 of 7 (14.3%) had a LMIC last author (Tables 3 and 4). These results mirror prior findings in the broader global health literature,^8^ where LMIC first authors were less frequently represented among articles published in high-impact journals, more likely to be published as first authors in journals with lower median impact factors compared with HIC first authors,^18^ and, among global surgical studies coming from LICs, more often produced work of lower-level evidence methods (case reports, series, and reviews).^19^ Prior work has also raised concerns regarding the power dynamics of HIC-LMIC partnerships and the inequities inherent in current academic research models, which may systematically favor collaborators from HICs at the expense of those from LMICs.^1,8,10,17^ Examples include definitions of authorship that devalue contributions from LMIC collaborators^10^ (which may be considered more technical and less deserving of authorship^17,18^) and the potential for editorial bias in favoring publication of articles from prominent authors from HICs.^17^ It is unclear whether any of the above factors can causatively explain our findings related to study the impact factor above, and future work will be needed to further explore publication patterns among LIC authors.

We found that studies which received funding were less likely to have LMIC last authors. Of LICs, 12 studies received funding, and of these, 3 (25%) had a LIC last author (Table 4). Prior authors have speculated on the causes of LMIC authorship underrepresentation with respect to funding, citing a lack of available grants or direct academic funding for LMIC authors,^6^ the location of most funding bodies based in HICs,^10^ and a virtuous cycle where HIC researchers advance their research agendas through prolific research facilitating funding acquisition.^9^ This discordance is particularly worrisome from a capacity building perspective. Strategic funding directly affects research agenda,^10^ and when local partners are sidelined in funding decisions, locally relevant research may be compromised.^10^ Future work will be needed to clarify whether any such underlying funding inequalities are at play in the orthopaedic literature.

We found global health–focused studies had some of the lowest levels of LMIC first authorship, 5 of 45 (11.1%), and last authorship, 8 of 44 (18.2%), of the study categories evaluated in our analysis (Table 1), and we observed global health studies and increasing multinationality of a study to be negative predictors for LMIC first and last authors (Table 5). Although our findings cannot definitively prove an underrepresentation of LMICs in global health studies, they are therein suggestive and, when considered in the context of the broader global health literature, consistent with previously raised concerns regarding low representation of local authors in global health studies,^7-9,19,20^ for many of the reasons already discussed above. Several solutions in other fields have been posited to change how local authors are represented and how their work is recognized. This may involve international journals redefining authorship or the types of articles being published to better represent local voices^21^ or seeking to include local discussions in the broader international discourse without demanding a conformation of local conversations to the traditional academic mold of peer-reviewed journals.^21^

Our study is not without limitations. We examined indexed journals, excluding nonindexed local journals. Although ostensibly multinational and multilingual in focus and scope, the journals indexed by PubMed and Web of Science largely represent journals based in HICs^22^ and may also exhibit an English language bias,^2^ which may introduce a selection bias for English language results and articles favoring HICs. Although these factors may also selectively exclude a subset of articles that may have higher rates of local authorship, this nonindexed literature is more difficult to find and access for a nonlocal audience and is thus somewhat separated from the global academic discourse. The indexed literature remains vitally important because it often helps inform policy decisions relating to international aid and future research.^2^ However, future work should assess the local literature in nonindexed journals to better characterize LMIC authorship. We chose to focus on first and last authorship in this analysis because these have been considered privileged positions on published studies, reflecting notable work or leadership in the completion of a study^1^; however, this convention may not be applicable in all cases or cultural settings. Further work will be needed to assess whether this convention can be fairly applied to all LIC and LMIC settings. Our analysis also excluded collaborative research involving LMIC authors occurring in HICs that do not pertain to LMICs, which may lead to an underrepresentation of the degree of multinational collaborative efforts involving LMIC authors. However, we chose to focus on the subset of collaborative work only pertaining to or occurring on LMICs, as we believed this would provide a clearer picture of international collaboration because it pertains to endemic research capacity and, ultimately, better investigate the dynamics governing the research in LMICs. In addition, we used author affiliation as a proxy for the country in which research is being conducted, which, although imperfect, represents the best available proxy given the bibliometric information provided and indicates at the very least affiliation with the scientific community of the indicated country.^2^ Furthermore, regarding authorship, although part of our analysis focused on first and last authors as key elements of research representation in our study, based on our methods, we are unable to fully assess the extent to which first and last authorship as identified in our study are truly representative of the amount of work and leadership that such positions imply for a study. However, the position of first and last authors still reflects the power dynamics governing the execution of a study and, as such, provides an informative perspective on how those power dynamics play out for LMIC authors on the studies included in this analysis. We did not stratify clinical studies in our analysis by study type or level of evidence, limiting our ability to assess the quality of studies from LICs and MICs in greater detail. Future work should consider doing so to develop a more robust understanding of research capacity in LMICs. Finally, we did not include orthopaedic articles published in nonorthopaedic journals, which may introduce some degree of selection bias that may warrant future exploration.

## Conclusion

In conclusion, our study represents one of the first to assess LMIC authorship in the global orthopaedic literature. The findings of our study help to provide an initial outline of this research landscape and underscore some of the ongoing challenges to equitable and representative research in low-income settings. Our study findings suggest the presence of disparities in LMIC authorship relating to funding, study type, journal impact factor, and degree of HIC collaboration and ultimately highlight some of the ongoing structural and systemic factors, which may hinder efforts to build local research capacity and support integration of local research efforts into broader global surgery and global orthopaedic research communities. Additional qualitative work is needed to help elucidate the potential inequities presented in this study.

## References

[1] Hedt-Gauthier BL Jeufack HM Neufeld NH : Stuck in the middle: A systematic review of authorship in collaborative health research in Africa, 2014-2016. BMJ Glob Health 2019;4:e001853. 10.1136/bmjgh-2019-001853 PMC6830050 31750000

[2] Cash-Gibson L Rojas-Gualdrón DF Pericàs JM Benach J: Inequalities in global health inequalities research: A 50-year bibliometric analysis (1966-2015). PLoS One 2018;13:e0191901. 29385197 10.1371/journal.pone.0191901 PMC5792017

[3] Iyandemye J Thomas MP Thomas MP: Low income countries have the highest percentages of open access publication: A systematic computational analysis of the biomedical literature. PLoS One 2019;14:e0220229. 31356618 10.1371/journal.pone.0220229 PMC6663019

[4] Kelaher M Ng L Knight K Rahadi A. Equity in global health research in the new millennium: Trends in first-authorship for randomized controlled trials among low-and middle-income country researchers 1990-2013. Int J Epidemiol 45:2174-2183. 10.1093/ije/dyw313 28199646

[5] Chu KM Jayaraman S Kyamanywa P Ntakiyiruta G: Building research capacity in Africa: Equity and global health collaborations. Plos Med 2014;11:e1001612. 24618823 10.1371/journal.pmed.1001612 PMC3949667

[6] Iyer AR: Authorship trends in the lancet global health. Lancet Glob Health 2018;6:e142. 29389534 10.1016/S2214-109X(17)30497-7

[7] Dimitris MC Gittings M King NB: How global is global health research? A large-scale analysis of trends in authorship. BMJ Glob Health 2021;6:e003758. 10.1136/bmjgh-2020-003758 PMC7843301 33500263

[8] Ghani M Hurrell R Verceles AC McCurdy MT Papali A: Geographic, subject, and authorship trends among LMIC-based scientific publications in high-impact global health and general medicine journals: A 30-month bibliometric analysis. J Epidemiol Glob Health 2020;11:92-97. 32959620 10.2991/jegh.k.200325.001 PMC7958272

[9] Pingray V Ortega V Yaya S Belizán JM: Authorship in studies conducted in low-and-middle income countries and published by reproductive health: Advancing equitable global health research collaborations. Reprod Health 2020;17:18. 32000792 10.1186/s12978-020-0858-7 PMC6993386

[10] Lawrence DS Hirsch LA: Decolonising global health: Transnational research partnerships under the spotlight. Int Health 2020;12:518-523. 33165557 10.1093/inthealth/ihaa073 PMC7651076

[11] Sgrò A Al-Busaidi IS Wells CI : Global surgery: A 30-year bibliometric analysis (1987-2017). World J Surg 2019;43:2689-2698. 31384996 10.1007/s00268-019-05112-w

[12] Graham SM Brennan C Laubscher M Orthopaedic research in low-income countries: A bibliometric analysis of the current literature. SICOT 2019;5:41 10.1051/sicotj/2019038 PMC6878915 31769752

[13] Clarivates. Journal impact factor - Journal Citation Reports - Web of Science group. https://clarivate.com/webofsciencegroup/solutions/journal-citation-reports/. Accessed December 18, 2021.

[14] World Bank Country and Lending Groups. World Bank data help desk. https://datahelpdesk.worldbank.org/knowledgebase/articles/906519-world-bank-country-and-lending-groups. Accessed April 9, 2022.

[15] Siegfried N Clarke M Volmink J: Randomised controlled trials in Africa of HIV and AIDS: Descriptive study and spatial distribution. BMJ 2005;331:742-746. 16195291 10.1136/bmj.331.7519.742 PMC1239977

[16] Aluede EE Phillips J Bleyer J Jergesen HE Coughlin R: Representation of developing countries in orthopaedic journals: A survey of four Influential Orthopaedic Journals. Clin Orthop Relat Res 2012;470:2313-2318. 22588702 10.1007/s11999-012-2377-5 PMC3392367

[17] Smith E Hunt M Master Z: Authorship ethics in global health research partnerships between researchers from low or middle income countries and high income countries. BMC Med Ethics 2014;15:42. 24885855 10.1186/1472-6939-15-42 PMC4061921

[18] Chersich MF Blaauw D Dumbaugh M : Local and foreign authorship of maternal health interventional research in low- and middle-income countries: Systematic mapping of publications 2000–2012. Glob Health 2016;12:35. 10.1186/s12992-016-0172-x PMC4917998 27338707

[19] Pauyo T Debas HT Kyamanywa P : Systematic review of surgical literature from resource-limited countries: Developing strategies for success. World J Surg 2015;39:2173-2181. 26037025 10.1007/s00268-015-3102-9

[20] Kohrt BA Upadhaya N Luitel NP : Authorship in global mental health research: Recommendations for collaborative approaches to writing and publishing. Ann Glob Health 2014;80:134-142. 24976552 10.1016/j.aogh.2014.04.007 PMC4155487

[21] Abimbola S: The foreign gaze: Authorship in academic global health. BMJ Glob Health 2019;4:e002068. 10.1136/bmjgh-2019-002068 PMC6830280 31750005

[22] Plancikova D Duric P O'May F: High-income countries remain overrepresented in highly ranked public health journals: A descriptive analysis of research settings and authorship affiliations. Crit Public Health 2020;31:487-493.

